# Kinetic Modeling for Photo-Assisted Penicillin G Degradation of (Mn_0.5_Zn_0.5_)[Cd*_x_*Fe_2-*x*_]O_4_ (x ≤ 0.05) Nanospinel Ferrites

**DOI:** 10.3390/nano11040970

**Published:** 2021-04-09

**Authors:** Omar Alagha, Noureddine Ouerfelli, Hafedh Kochkar, Munirah A. Almessiere, Yassine Slimani, Ayyar Manikandan, Abdulhadi Baykal, Ahmed Mostafa, Mukarram Zubair, Mohammad H. Barghouthi

**Affiliations:** 1 Environmental Engineering Department, College of Engineering, Imam Abdulrahman Bin Faisal University, Dammam 31441, Saudi Arabia; mzzubair@iau.edu.sa (M.Z.); mhbarghouthi@iau.edu.sa (M.H.B.); 2 Department of Chemistry, College of Science, Imam Abdulrahman Bin Faisal University, Dammam 31441, Saudi Arabia; Nouerfelli@iau.edu.sa (N.O.); hbkochkar@iau.edu.sa (H.K.); 3 Basic & Applied Scientific Research Center, Imam Abdulrahman Bin Faisal University, Dammam 31441, Saudi Arabia; 4 Department of Biophysics, Institute for Research & Medical Consultations (IRMC), Imam Abdulrahman Bin Faisal University, Dammam 31441, Saudi Arabia; malmessiere@iau.edu.sa (M.A.A.); yaslimani@iau.edu.sa (Y.S.); 5 Department of Chemistry, Bharath Institute of Higher Education and Research (BIHER), Bharath University, Chennai 600073, India; manikandana.che@bharathuniv.ac.in; 6 Department of Nanomedicine Research, Institute for Research & Medical Consultations (IRMC), Imam Abdulrahman Bin Faisal University, Dammam 31441, Saudi Arabia; abaykal@iau.edu.sa; 7 Department of Pharmaceutical Chemistry, College of Clinical Pharmacy, Imam Abdulrahman Bin Faisal University, Dammam 31441, Saudi Arabia; ammostafa@iau.edu.sa

**Keywords:** nanoparticles, photodegradation, penicillin, kinetic modeling, mixed spinel ferrites, wastewater, emerging pollutants

## Abstract

Penicillin G is an old and widely used antibiotic. Its persistence in the environment started to appear in many environmental samples and food chains. The removal of these emerging pollutants has been a challenging task for scientists in the last decades. The photocatalytic properties of Cd^2+^ doped Manganese- Zinc NSFs with chemical formula (Mn_0.5_Zn_0.5_)[Cd*_x_*Fe_2−*x*_]O_4_ (0.0 ≤ x ≤ 0.05) NSFs are herein evaluated. The Manganese- Zinc N.S.F.s nanomaterials were deeply characterized, utilizing UV-Vis (reflectance) spectroscopy, X-ray diffraction, N_2_ adsorption isotherm measurements, and S.E.M., SEM-EDX mapping, and T.E.M. The Kinetic model for the photodegradation of penicillin G (as a model molecule) is investigated using visible light as a source of energy. The kinetic study shows that our results fit well with the modified pseudo-first-order model. The Pen G degradation are 88.73%, 66.65%, 44.70%, 37.62% and 24.68% for x = 0.5, 0.4, 0.3, 0.2 and 0.1, respectively, against 14.68% for the free Cd spinel sample. The pseudo-rate constant is bandgap dependent. From the intra-diffusion rate constant (K_d_), we developed an intra-diffusion time (τ) model, which decreases exponentially as a function of (x) and mainly shows the existence of three different domains versus cadmium coordination in spinel ferrite samples. Hence, Cadmium’s presence generates spontaneous polarization with a strong opportunity to monitor the charge separation and then open the route to a new generation of “assisted” photocatalysts under visible light.

## 1. Introduction

Nowadays, the contamination of water bodies due to the presence of pharmaceutical drugs cause a devastating effect on the environment and is considered to be a serious concern worldwide. Among them, antibiotics are widely used medicines to treat human and veterinary infections [1]. A human generally consumes antibiotics for the treatment of bacterial diseases, and their primary usage in animal and agriculture farming is for the prevention of disease growth. For instance, the annual use of antibiotics was nearly 1.62 million, 13 and 10 thousand tons in China, the U.S.A., and Europe, respectively. The active antibiotic is detected in the water bodies (surface, ground, and industrial) and soil due to the discharge released from pharmaceutical effluents as well as domestic and agricultural waste. The commonly used antibiotics remain approximately 90% active after usa [2]. Therefore, their presence in the environment can adversely impact the water quality and eco-system [3,4,5,6,7].

Penicillin G is a commonly used antibiotic with diverse applications in treating infections associated with susceptible bacteria or other related diseases [8]. Penicillin G is a water-soluble antibiotic, and its general mechanism is to degrade the structure of the bacteria cell, which prevents the formation of peptidoglycan [9]. Conventional treatment methods for removing antibiotics such as filtration, reverse osmosis, adsorption, and bioremediation are found to be ineffective. The advanced oxidation process (AOP) is considered to be an environmentally friendly and highly efficient technique for the degradation of antibiotic pollutants. The AOP accelerates the degradation of a wide range of pharmaceutical pollutants that are difficult to treat by conventional methods [10,11,12]. In AOP, the contaminants are degraded or converted into low molecular weight molecules, improving biodegradability and removal efficiency. This is due to the generation of highly reactive oxygen species such as hydroxyl radical (OH°), ozone (O_3_), and super oxide radical (O_2_^-^°), which facilitate the oxidation of antibiotics and transform them into harmless by-products [13]. Photocatalytic degradation has been widely studied for the decomposition of pharmaceutical contaminants. The most commonly studied photocatalyst are TiO_2_, Zn_S_, SnO_2_ and WO_3._ These are semi-conductor materials that excite due to the absorption of energy. This generates superoxide radical (°O_2_^-^) and hydroxyl radical (°OH^-^) due to the oxidation and reduction reaction and could degrade antibiotics.

In the last decade, the application of nanoparticles in water purification technologies has gained enormous attention. The large surface area, chemical and thermal stability, abundant functionalities, and high light activity confirm them as potential photocatalyst for the treatment of various antibiotics from water streams. For example, Au/Pt interacted g-C_3_N_4_ showed excellent photocatalytic degradation of antibiotic tetracycline hydrochloride, which is 3.4 times more than that of g-C_3_N_4_ [14]. Besides, magnetic nanoparticles and their derivatives are attractive as an ideal nanomaterial as photocatalyst in wastewater treatment, reducing the effort and cost of catalyst separation and better reusability after several treatment processes [15]. For example, magnetic recoverable MnFe_2_O_4_ decorated graphitic carbon sand catalyst exhibited a robust degradation of ampicillin and oxytetracycline antibiotics. The catalyst’s performance was effective for up to 10 reusability cycles [16]. Recently, Hangdao Qin et al. reported in their study the highest catalytic activity of MnFe_2_O_4_@carbon-NH_2_ towards tetracycline, amoxicillin, and ofloxacin [17]. Previous studies revealed that trimetallic nanoparticles exhibited substantially improved electrocatalytic performance than Pt-based trimetallic nanoparticles supported with carbon [18]. Microwave prepared Trimetallic nanoparticles La/Cu/Zr, was investigated for the degradation of ampicillin and showed up to 86% decomposition of antibiotic [19]. Therefore, it was expected that metallic ions into trimetallic nanoparticles might lead to significant enhancement in the nanoparticle’s catalytic performance. Based on a detailed literature review, no work has been reported as investigating the doping of different mole ratios of Cd onto MnZnFe trimetallic nanoparticles.

The main objective of this work is to evaluate the photocatalytic properties of Cd^2+^ doped Manganese- Zinc N.S.F.s with empirical formula (Mn_0.5_Zn_0.5_)[Cd*_x_*Fe_2-*x*_]O_4_ (x ≤ 0.05) NSFs onto penicillin G antibiotic utilizing visible light as an energy source. 

## 2. Materials and Methods

### 2.1. Elaboration of (Mn_0.5_Zn_0.5_)[Cd_x_Fe_2−x_]O_4_ NSFs

Cd^2+^ doped Manganese- Zinc NSFs with empirical formula (Mn_0.5_Zn_0.5_)[Cd*_x_*Fe_2−*x*_]O_4_ (*x ≤* 0.05) NSFs were produced by the UV irradiation method. A proper ratio of Fe[NO_3_]_3_.9H_2_O (Iron Nitride), Zn[NO_3_]_2_ (Zin Nitride), Cd[NO_3_]_2_.4H_2_O (Cadmium Nitride), and MnCl_2_.4H_2_O (Manganese Chloride) were dissolved in 100 mL of Deionized water. After stirring the solution continuously for one h, the pH was amended at 11 by drop wisely of NaOH. Subsequently, the final solution was exposed to the U.W. irradiation for 40 min via 70 W and 20 kHz Ultrasonic Homogenizer from UZ SONOPULS HD 2070. The resulting mixture was washed 3 to 5 times with Deionized hot water and then dried overnight at 60 °C.

### 2.2. Photocatalytic Test

Photocatalytic tests were performed using an aqueous solution (30 cm^3^) containing Penicillin G (PenG, 100 mg/L) as a model contaminant. The reaction was carried out in a double-walled thermostat Pyrex photoreactor (100 cm^3^) with an optical window area of 12.5 cm^2^. The concentration of photocatalyst was set at 1.0 g.L^−1^. The pH of the different solutions was close to 6.0 ± 0.2 and the temperature was set as constant with chiller at 20.0 ± 0.2 °C for all the experiments. HQI-E 400 W/n plus visible lamp coated with UV filter was used for visible irradiation. The rated lamp efficacy in the standard condition is 91 lm/W; the filter coats the lamp for UV radiation removal Typically, in a photocatalytic experiment, 30 mg of (Mn_0.5_Zn_0.5_)[Cd*_x_*Fe_2-*x*_]O_4_ (*x ≤* 0.05) NSFs ultrasonically suspended in 30 mL of 100 ppm aqueous solution of contaminant (PenG). Stirring the mixture for 60 min to reach the adsorption equilibrium in the dark; then, while the mixture was air bubbled and stirred, it was photo-irradiated at 20.0 ± 0.2 °C, utilizing a solar lamp (HQI-E 400 W/n plus visible lamp). After reaching equilibrium, the substrate concentration was measured, and considered the initial concentration (C0) to deduct the dark adsorption. PenG samples were taken at different intervals (C_t_) from the reactor’s solution upper part, and then filtered using 0.45 µm nylon syringe filters. Consequently, the samples were analyzed utilizing a Shimadzu High-Performance Liquid Chromatography (HPLC) and (300 mm × 7.8 mm) Hypersil Gold column and a U.V.–Vis detector (*λ* = 210 nm). A mobile phase of H_2_SO_4_ (510^−^^3^ mol/L) was used, flowing at 1 mL/min. During all the experiments, three runs for all the photocatalytic tests have been achieved with good reproducibility. 

In this work, the problem of diffusion-controlled kinetics is studied by using a modified diffusion model for Pen G photodegradation in the batch reactor. Here, the variation of PenG concentration vs. time was used for this model. We demonstrated that $\ln\left(\frac{C}{C_{0}}\right)=\;-\;\sqrt{\frac{t}{\tau }}$ is defined as the diffusion time. This model fits well with the photodegradation of PenG. We demonstrated that a high degradation rate is obtained at a low value. In addition, this diffusion factor decreases exponentially versus Cd (x) in the Mn_0_._5_Cd_1.5x_Fe_2−x_O_4_ catalysts. Finally, this model can be used to predict the catalytic behavior versus Cd loading.

### 2.3. Catalysts Characterization

Phase identification was implemented by the (Rigaku D/Max-IIIC, Tokyo, Japan) XRD system with Cu Kα radiation. The microstructure was analyzed via (JEOL JSM-6490, Pleasanton, USA) scanning electron microscopy (S.E.M.) coupled with E.D.X. T.E.M., SAED patterns, and high-resolution T.E.M. (HR-TEM) analyses were performed using an F.E.I. Titan S.T. microscope (300 keV). Pore structure and surface area measurements were performed using a Micrometrics ASAP 2020 instrument, Norcross, USA. Before the adsorption measurement, 0.05 g of the calcined catalyst was degassed by flowing nitrogen for 3 h at 240 °C. The adsorption isotherms were plotted at 196 °C (liquid nitrogen temperature). Relying on the B.J.H. adsorption calculation method, pore diameter, pore-volume, and pore surface area were measured.

## 3. Results and Discussion

### 3.1. Analysis of Phase and Morphology 

**Figure 1:**
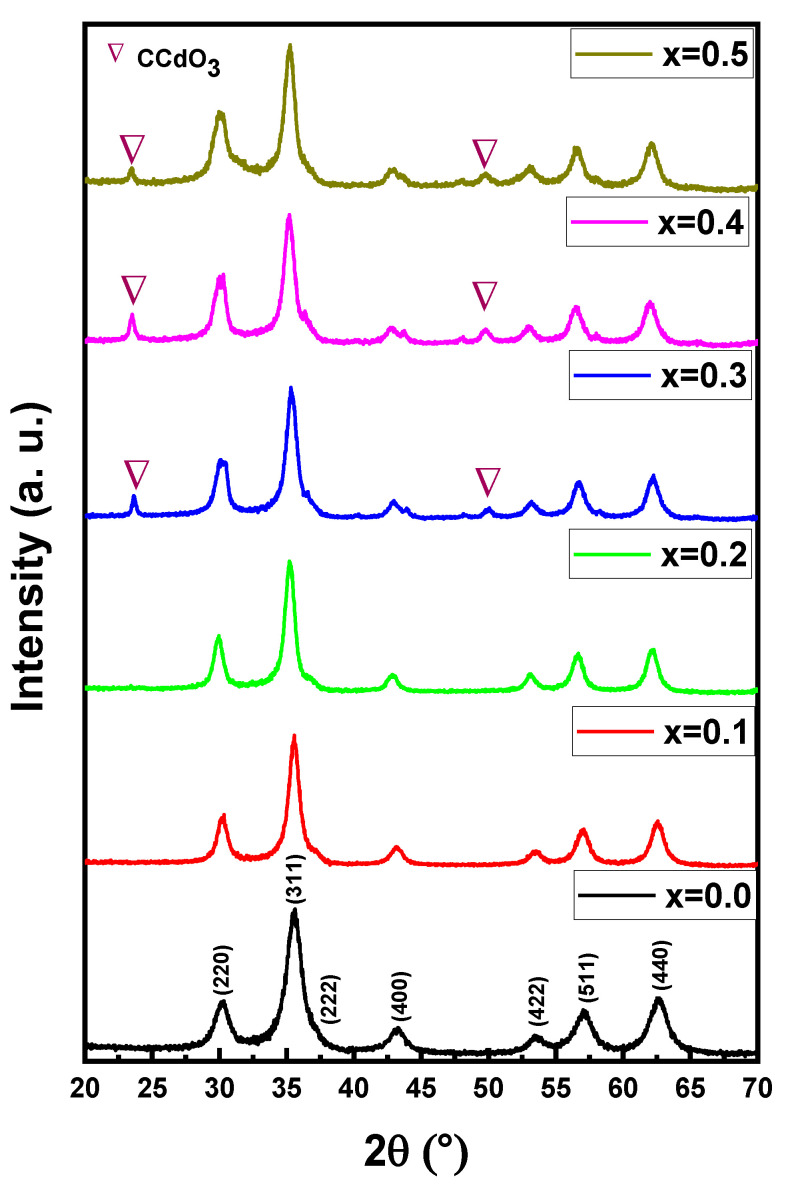
XRD powder patterns of (Mn_0.5_Zn_0.5_)[Cd*_x_*Fe_2−*x*_]O_4_ (x ≤ 0.05) NSFs.

**Figure 2:**
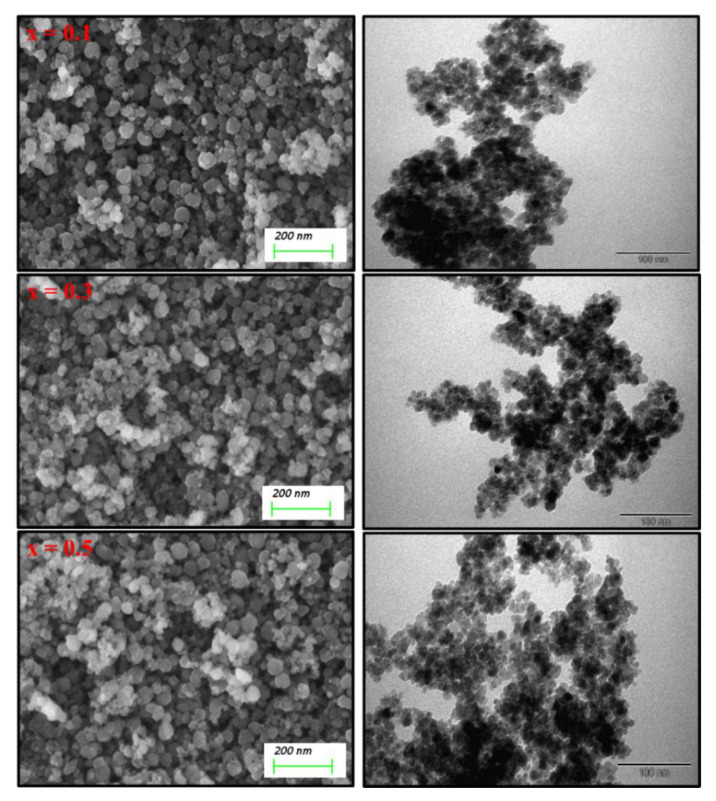
SEM and TEM images of (Mn_0.5_Zn_0.5_)[Cd*_x_*Fe_2−*x*_]O_4_ (x = 0.1, 0.3 and 0.5) NSFs.

**Figure 3:**
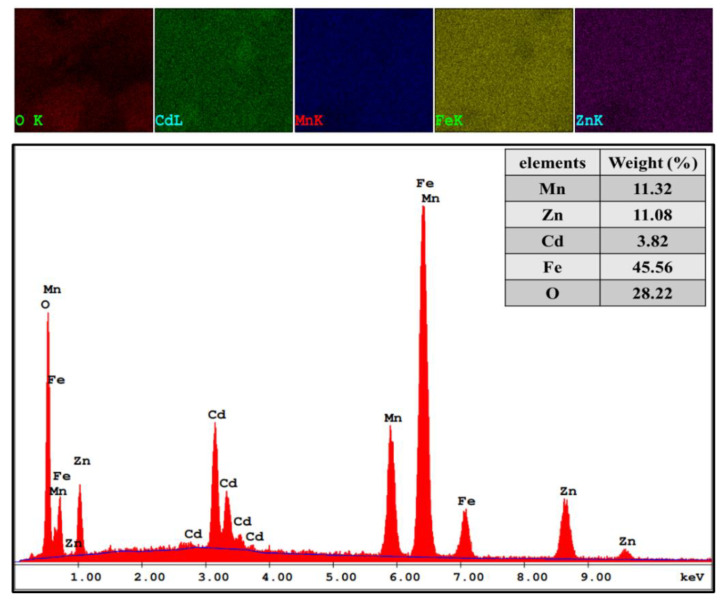
EDX and elemental mapping of (Mn_0.5_Zn_0.5_)[Cd*_x_*Fe_2−*x*_]O_4_ (x = 0.2) NSFs.

Figure 1 exhibitions of the X-ray powder of (Mn_0.5_Zn_0.5_)[Cd*_x_*Fe_2-*x*_]O_4_ (x ≤ 0.05) NSFs. The characteristic peaks of Manganese- Zinc spinel ferrite structure with space group Fd3m appeared in all samples. There is a miner phase of CCdO_3_ at x = 0.3 to 0.5. The lattice constant ‘*a’* was increased by raising the Cd ratio in the range of 8.312(5)–8.508(0). Further, the average crystal size was evaluated using the Debye-Scherrer formula and was found to be in the range of 6–9 nm. Figure 2 demonstrates the TEM and FE-Scanning Electron Microscope images of (Mn_0.5_Zn_0.5_)[Cd*_x_*Fe_2-*x*_]O_4_ (x = 0.1, 0.3 and 0.5) NSFs. The intermediate magnification images indicated a cluster of small, homogeneously distributed semi-cubic particles. The efficiency of the preparation method was approved through elemental mapping and EDX spectra of (Mn_0.5_Zn_0.5_)[Cd*_x_*Fe_2-*x*_]O_4_ (x = 0.2) NSFs, as seen in Figure 3. It showed the weight percentage of consistent elements like Mn, Zn, Cd, Fe, and O. 

### 3.2. Nitrogen Physisorption

In order to gain more insights into the textural properties of the prepared materials, the surface area analysis was carried out using the TriStar II PLUS surface area analyzer from Micromeritics, Norcross, USA, which was used to obtain measurements of pore size distribution, pore-volume, and surface area of the samples. The materials were degassed at 190 °C for three hours under a vacuum in order to eliminate impurities prior to N_2_ physisorption measurements. 

**Figure 4:**
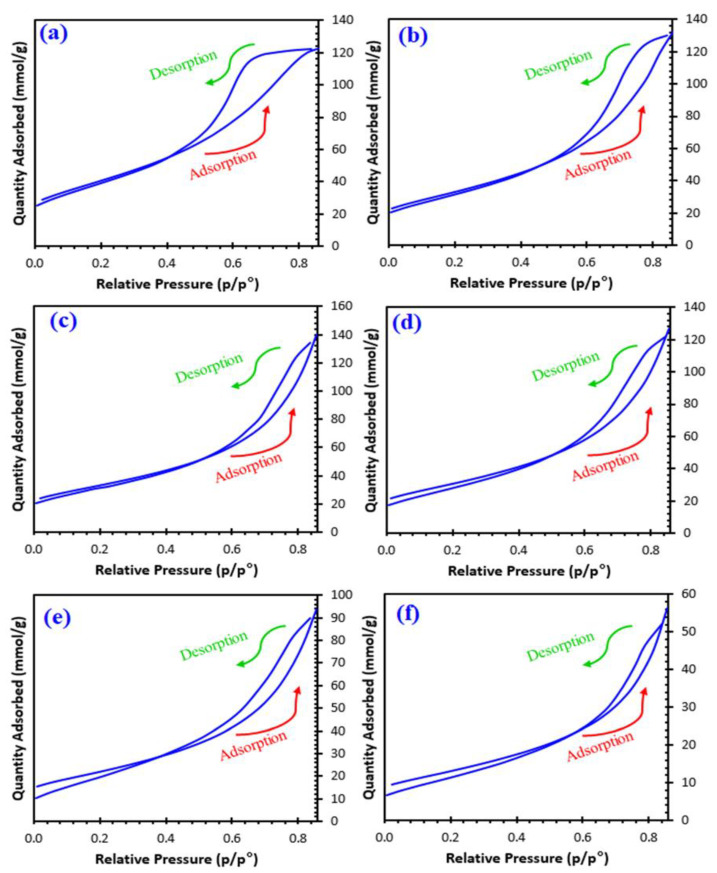
Nitrogen physisorption isotherm plots of; (**a**) x0, (**b**) x01, (**c**) x02, (**d**) x03, (**e**) x04, and (**f**) x05.

Table 1 lists the surface area, pore volumes, and pore size distribution of the samples, including the isotherm multipoint B.E.T. surface area and Langmuir surface area. Figure 4 depicts the sorption isotherm curves and reflects the relationship between gas sorption and porosity. The shape of isotherm can be considered under types II and III. The pore size is commonly defined as the distance between two opposite walls or as the pore width. Precise pore size indicates that the geometrical shape is evident. The porosity of any material is defined as the ratio of the volume of voids and pores to the volume the solid occupies. Adsorption properties of the material also determine its porosity. As listed in Table 1, the pore size ranges between 2 and 50 nm, indicating a mesoporous nature. It should be noticed that the size decreases from sample 1 to sample 5, indicating possible mesoporous type materials. 

The more complex pore structures resulted in the materials x = 0 and x = 0.1 being close to type H2 where the network effects are significant. According to IUPAC, the other materials show the adsorption hysteresis H3, indicating that the materials’ pores are slit-shaped (the isotherms exhibiting type H3 do not limit adsorption high P/Po ratio, which is detected in flexible aggregates of platelike particles). H3 hysteresis desorption curves contain slopes associated with a force on the loop of the hysteresis, owing to what is called the tensile strength effect [20,21,22]. 

### 3.3. Optical Properties

The optical properties of (Mn_0.5_Zn_0.5_)[Cd*_x_*Fe_2-*x*_]O_4_ (0.0 *≤ x ≤* 0.05) NSFs were investigated using a diffuse reflectance UV-vis spectrometer. Figure S1 (Supplementary Information) shows the percent diffuse reflectance (D.R. %) data recorded in the 200 nm–800 nm in the UV-Vis range. Energies range from 1.55 eV to 6.2 eV corresponds to the entire sweep spectral region. The range of the spectra reflectance intensities starts from a narrow band between 13% to 15% for the first part of the entire sweep spectral region (200 nm to 500 nm). At all reflectance intensities, sharp increases were observed up until a maximum at 45% for the rest of the sweep range. To evaluate the diffuse reflectance spectra, the Kubelka-Munk (K-M) function $F\left(R_{\infty }\right)$ is frequently used

(1) $$F\left(R_{\infty }\right)=\frac{{\left(1-R_{\infty }\right)}^{2}}{2R_{\infty }}=\frac{K}{S}$$

This function connects the abstract absorption quantity *K* to diffuse reflectance $R_{\infty }$ and scattering quantity *S*. The $F\left(R_{\infty }\right)$ is designed to calculate the *K*/*S* ratio; extra information about the absorption characteristics can also be obtained. The K-M function becomes dependent on *K* alone, assuming an unremarkable change of *S* falls in the wavelength range of electronic absorption. In this way, the absorption α, which is related to *K*, can be extracted via a functional relation covering the energy of the incident photon E=hυ and the electronic transition-dependent exponent *n* [23]:

(2) $${\left(F\left(R_{\infty }\right)∙h\upsilon \right)}^{n}\sim {\left(\alpha .h\upsilon \right)}^{n}$$

**Figure 5:**
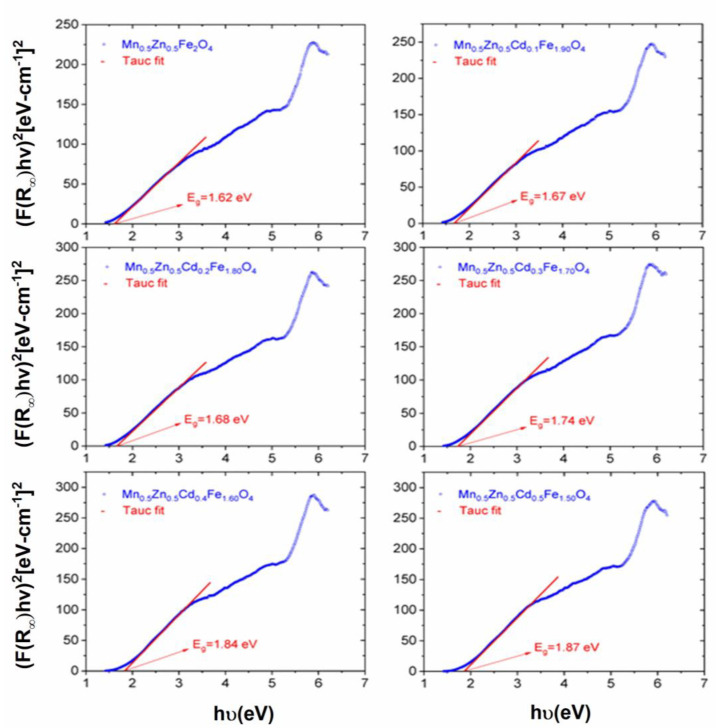
Tauc plots of (Mn_0.5_Zn_0.5_)[Cd*_x_*Fe_2-*x*_]O_4_ (x ≤ 0.05) NSFs. Extrapolating the straight portion of the plot to the photon energy axis at the 〚(αhυ)〛^2 = 0 determines the value of the optical band gap.

Linear correlation between optical absorption and optical energy band gap (*E*_g_) of samples are given by the expression:

(3) $$\alpha ∙h\upsilon =A_{1}{\left(h\upsilon -E_{g}\right)}^{n}$$

where $A_{1}$ is an arbitrary proportionality constant, a combination of Equations (2) and (3) relates the *E*_g_ and $F\left(R_{\infty }\right)$ and is also known as the Tauc Equation [24]:

(4) $${\left(F\left(R_{\infty }\right)∙h\upsilon \right)}^{1/n}=A_{2}\left(h\upsilon -E_{g}\right)$$

where $A_{2}\;$ is the proportionality constant, and *(n* = 1/2) directly symbolizes the allowed electronic transition. By plotting ${\left(F\left(R_{\infty }\right)h\upsilon \right)}^{2}$ versus photon energy (hυ) graphs, *E*_g_ values can be estimated. A straight line fit to the linear section of the graph intercepts with the energy (y) axis at ${\left(F\left(R_{\infty }\right)h\upsilon \right)}^{2}=0$. After that, the corresponding value on the energy (y) axis is assigned as *E*_g_ in eV units. Figure 5 presents all Tauc fits and estimated band gaps belonging to our samples. Mixed spinel Mn_0.5_Zn_0.5_Fe_2_O_4_ has a 1.62 eV *E*_g_ value. Cd^3+^ ion coordinated samples have band gaps 1.67, 1.68, 1.74, 1.84 and 1.87 eV of magnitude, corresponding to increasing Cd^3+^ ion proportion from x = 0.1 to x = 0.5. Hence, it is observed that the coordination of Cd^3+^ ion causes significant increments at direct *E*_g_ of mixed spinel ferrite sample. However, all estimated *E*_g_ data are in the bandgap range of semiconductors. Ashok and Nam’s groups report the direct *E*_g_ values of 1.98 and 1.99 eV for mixed Mn_0.5_Zn_0.5_Fe_2_O_4_ NPs, which were prepared via hydrothermal and sonication assisted microwave irradiation methods, respectively [25,26]. Our group reported the bandgap data of Mn_0.5_Zn_0.5_Fe_2_O_4_ and Mn_0.5_Zn_0.5_Dy_x_Fe_2-x_O_4_ (*x* = 0.01–0.03) NPs were produced using ultrasonic irradiation in a narrow range from 1.61 to 1.67 eV [27]. However, there have not been any reported *E*_g_ data for (Mn_0.5_Zn_0.5_)[Cd*_x_*Fe_2-*x*_]O_4_ NSFs in the literature. 

### 3.4. Kinetic Study

**Figure 6:**
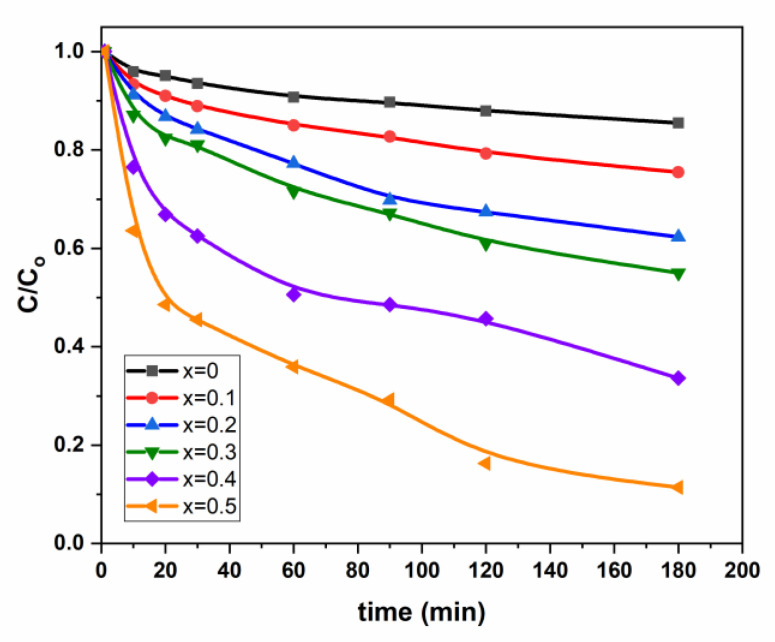
Variation of C/C_0_ vs. time for (Mn_0.5_Zn_0.5_)[Cd*_x_*Fe_2−*x*_]O_4_ (*x =* 0.1, 0.3 and 0.5) NSFs.

The photodegradation of Pen G onto (Mn_0.5_Zn_0.5_)[Cd*_x_*Fe_2-*x*_]O_4_ (*x* ≤ 0.05) NSFs are herein evaluated under visible light radiation. Table 2 and Figure 6 summarize these results. The photolysis test of PenG under visible light shows low conversion (<4.4 %) within the 3 h test. Thus, the photodegradation of PenG without the catalyst can be neglected since higher conversions are obtained with Mn_0_._5_Cd_1.5x_Fe_2-x_O_4_ 14.88% (*x*0), 24.68 %(*x*0.1), 37.62%(*x*0.2), 44.70%(*x*0.3), 66.65%(*x*0.4) and 88.73% (*x*0.5).

Table 2 and Figure 6 present the variation of C_0_/C as a function of cadmium coordination (*x)*. The first overview of the curvatures’ trend indicates that the degradation is enhanced by increasing Cd coordination in mixed spinel Mn_0.5_Zn_0.5_Fe_2_O_4_ catalysts (Figure 6). The Pen G degradation are 88.73%, 66.65%, 44.70%, 37.62% and 24.68% for *x* = 0.5, 0.4, 0.3, 0.2 and 0.1, respectively, against 14.68% for free Cd spinel sample. Herein, we might have a Cadmium “assisted” Pen G photodegradation due to decreasing the photogenerated (electron-hole) pairs recombination. 

The photodegradation rate of Pen G can be expressed as

(5) $$\mathrm{Rate}\;=\;-\frac{d\;\left[Pen\;G\right]}{dt}\;=\;k_{i}\;[\mathrm{Pen}\;G{]}^{a}\;(\mathrm{at}\;\mathrm{constant}\;\mathrm{temperature})$$

If we consider that the reaction follows pseudo first order kinetics, then a = 1, and thus the equation (Equation (5)) becomes:

(6) $$\mathrm{Rate}\;=\;-\frac{d\;\left[Pen\;G\right]}{dt}\;=\;k_{i}\;[\mathrm{Pen}\;G]\;$$

By rearranging equation (Equation (6)) we obtain:

(7) $$\mathrm{Rate}\;=\;\frac{d\;{\left[Pen\;G\right]}_{\;}}{{\left[\mathrm{Pen}\;G\right]}_{\;\;}}\;=\;-k_{1}\;\mathrm{dt}$$

By integrating the expression (Equation (7)) from the t = 0 at initial concentration to t > 0 at final concentration, the Equation (7) becomes:

(8) $$\int_{c_{0}}^{c_{t}}\frac{d\;Pen\;G_{}}{{\left[\mathrm{Pen}\;G\right]}_{}}=-\;\overset{t}{\underset{0}{\int }}\;k1\;dt\;\;$$

where [Pen G]_0_ is Penicillin G concentration at time zero = C_0_, and [Pen G]_t_ is the concentration at time (t > 0) C _t._

(9) $$\mathrm{Ln}\left(\frac{{\left[c\right]}_{t}}{{\left[c\right]}_{0}}\right)\;\;=\;-k_{1}\;t$$

**Figure 7:**
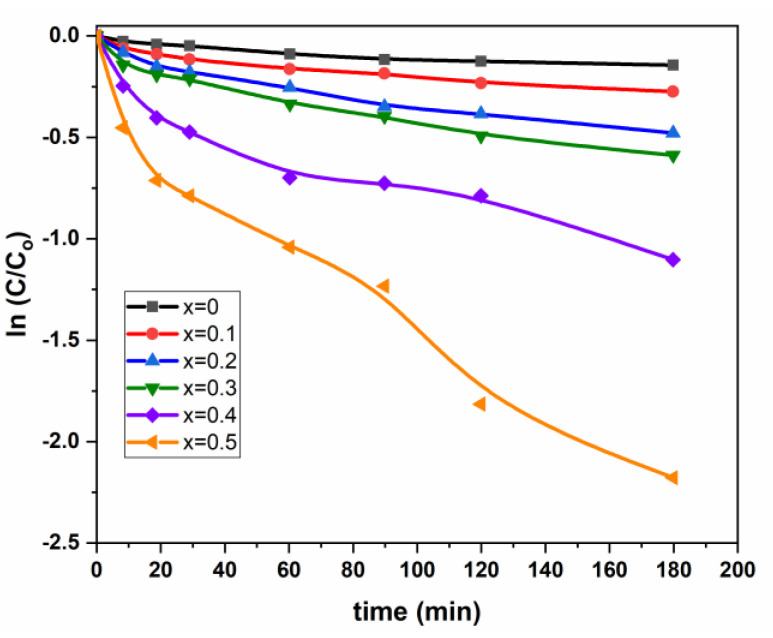
Variation of ln C/C_0_ vs. time for (Mn_0.5_Zn_0.5_)[Cd*_x_*Fe_2-*x*_]O_4_ (x = 0.1, 0.3 and 0.5) NSFs.

Therefore, we can get the rate constant by plotting $\mathrm{Ln}\left(\frac{{\left[c\right]}_{t}}{{\left[c\right]}_{0}}\right)$ vs. time; the slope gives the value of pseudo first-order rate constant. Table 3 and Figure 7 summarize these results.

**Figure 8:**
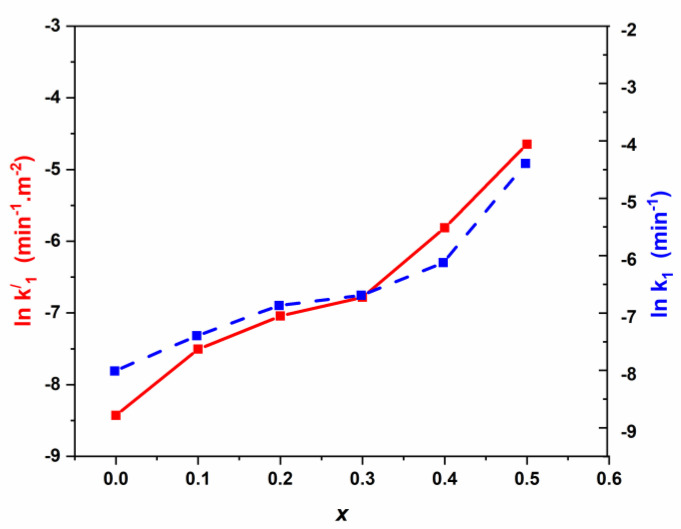
Variation of ln k1 and lnk1’ vs. cadmium coordination in NSFs.

From Table 3, we can undertake the kinetic model for Pen G photodegradation using Cd modified Mn_0.5_Zn_0.5_Fe_2_O_4_ spinel catalysts assigned to pseudo first-order. From Table 2, it seems that the pseudo first-order rate constant k_1_ (min^−1^) increases versus Cd loading. Nevertheless, from N_2_ adsorption-desorption results, the specific surface areas of Mn_0.5_Zn_0.5_Fe_2_O_4_ spinel catalysts decreased tremendously vs. Cd loading (the SBET is 138 m^2^·g^−1^ for Mn_0.5_Zn_0.5_Fe_2_O_4_ against 43 m^2^·g^−1^ for Cd (x = 0.5) Mn_0.5_Zn_0.5_Fe_2_O_4_)_._ The amount of catalyst was kept constant for all the experiments, and to elucidate the effect of the variation of surface area, the pseudo rate constants can be expressed per m^2^ (the equivalent to the intrinsic activity k_1_’). The natural logarithm ln(k_1_) and ln(k’_1_) were plotted vs. Cd loading for the different spinel catalysts (Figure 8). 

From Figure 8, similar trends are obtained for simultaneously the pseudo rate constant (lnk_1_) and intrinsic rate (lnk’_1_), showing no significant effect on the surface area. Moreover, the kinetic process seems to be more affected by cadmium loading (x). Three domains are mainly obtained, namely: (I) for *x* < 0.2, *k*_i_ increases linearly; (II) for 0.20 < *x* < 0.30, *k*_i_ increases slowly and (III) for *x* > 0.30, *k*_i_ increases linearly. 

Figure 7 shows that the small deviation to the linearity; this feeble deviation to the first pseudo order leads us to think about a process with a probable optimal pseudo average order k_n_ (n > 1 and n ≠ 1) for correlating the present degradation (Equation (10)).

(10) $$\frac{C}{C_{0}}={\left[\left(n-1\right)k_{n}t+1\right]}^{-\frac{1}{n-1}}$$

where the intermediate pseudo order is determined by nonlinear regression, and it is approximately (*n* = 1.65). Nevertheless, the few values of (C/C_0_) and the lack of smoothness in Figure 7 can significantly affect the precedent *n*-value. For this constraint, and to reduce the discrepancy between experimental and estimated values, we thought it better to extend the model of the first pseudo order (Equation (9)) by a small modification in the second number, which becomes a second-degree polynomial by adding a *t*^2^-term, and this was expressed as follows:

(11) $$\ln\left(\frac{C}{C_{0}}\right)=-a_{1}t+a_{2}t^{2}$$

In order to compare the first pseudo-order (Equation (9)) with the proposed modified one (Equation (11)), parameter (*a*_1_) has been put in the common factor (Equation (12)), which becomes equivalent to a kinetic rate constant where the new derived parameter (α) represents an increment and its amount shows how much the kinetic process deviates from the true pseudo first-order.

(12) $$\ln\left(\frac{C}{C_{0}}\right)=-a_{1}t\left(1-\alpha t\right)$$

Table S1 (Supplementary Information) presents values of the new adjustable parameters and the corresponding correlation coefficient (*R*), which exhibit a clear improvement. In addition, the values of (α) decrease with the increase of (*x*), showing that the nature of the kinetic process approaches the true first pseudo order for high values of *x*. We note that this ascertainment is also confirmed by the R-values increase in Table 3 related to the kinetic rate constant (*k*_1_).

**Figure 9:**
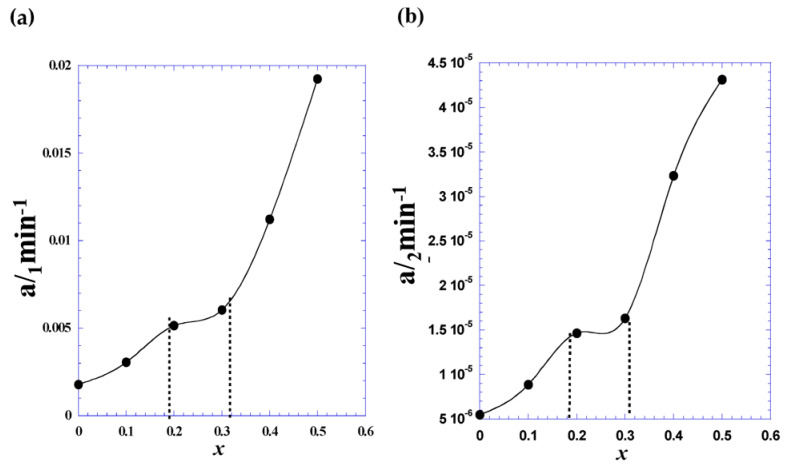
The variation of the adjustable parameters a1 (**a**) and a2 (**b**) vs. Cd coordination (x).

Figure 9 shows the variation of the adjustable parameters *a*_1_ (Figure 9a) and *a*_2_ (Figure 9b) vs. Cd coordination (*x).* We noticed the presence of three domains with distinct behaviors, which confirms the earlier observation in Figure 8. However, the corrected rate constant (*a*_1_) shows a clear jump for x higher than 0.3. 

Zhou et al. [28] investigated the kinetic simulation for U.V./peroxydisulfate penicillin removal and the degradation mechanism. The direct photolysis of Pen G in the UV 254 nm line was substantial and followed the kinetics of the pseudo first-order rate constant of 1.2710^−3^ s^−1^ (0.02 10^−3^ min^−1^). The same authors found that the U.V./peroxodisulfate (P.D.S.) process enhanced the photodegradation rate of Pen G; a pseudo rate constant of 0.5 × 10^−3^ min^−1^ is achieved in the presence of 5 mM P.D.S. In the present work, mixed spinel ferrite (x = 0.5) shows a pseudo-rate constant of 12.47 × 10^−3^ min^−1^ (~25 times higher) under visible light. Moreover, the drawback of the U.V./peroxodisulfate (P.D.S.) process is sulfate ions formation.

Navarra et al. [29] demonstrated that the U.V./Zn^2+^ system is effective in the photodegradation of four classical penicillins: ampicillin, amoxicillin, and both G&V penicillins. The pseudo-rate constant for penicillin G is 0.349 × 10^−3^ min^−1^. Using the Cd^2+^/UV system, the pseudo-rate constant for penicillin G reached 0.79 × 10^−3^ min^−1^ [30]. Using transition metals U.V. system, it has been proposed that catalysis occurs via an intermediate 1:1 complex. This mechanism is formed between the metal ion and the antibiotic, where the role of the metal ion in aminolysis or hydrolysis is to establish the tetrahedral intermediate, which is formed when the nucleophilic group is added to the b-lactam carbonyl group [31,32]. Mohammad Kamranifar et al. [33] evaluated CoFe_2_O_4_@CuS magnetic nanocomposite’s efficiency in photocatalytic degradation of Pen G in aqueous solutions, a pseudo first-order of 0.6 × 10^−3^ min^−1^ is reached. Based on the cited examples, mixed spinel ferrite catalysts (x = 0.1, 0.2, 0.3, 0.4, and 0.5) show the highest photodegradation rates for penicillin G under visible light.

### 3.5. Intra-Diffusion Study

**Figure 10:**
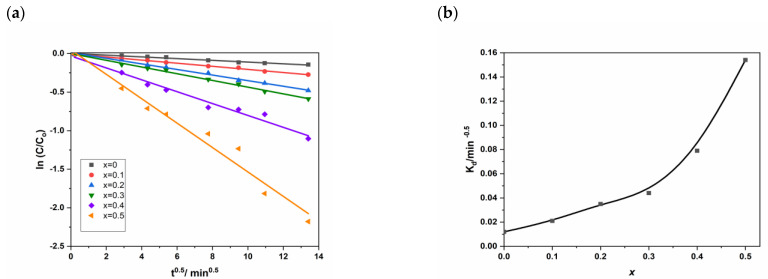
(**a**) ln(C/C0) for different as a function t1/2 and (**b**) Kd vs. cadmium coordination in NSFs.

Figure 10a represents the natural logarithm ln(*C*/*C*_0_) for different values of *x* as a function of the square root of time (*t*^1/2^). We can observe a clear quasi-linearity (Equation (13)) confirmed by the high values of the correlation coefficient (*R*) in Table S2 (Supplementary Information).

(13) $$\ln\left(\frac{C}{C_{0}}\right)=-K_{d}\times \sqrt{t}$$

Figure 10b shows a global increase of the intra-diffusion rate constant (*K_d_*) with Cd loading (*x*) and confirms the previous ascertainments by the kinetic order mentioned above. Besides, we see that the two first domains have similar behavior, while for the third domain (*x* > 0.30) we observe an accentuation of the phenomenon.

To provide the possible physical meaning on the intra-diffusion rate constant (*K_d_*), Equation (13) was rewritten into a new expression (Equation (14)) where the new parameter (*τ*) designates the characteristic time of the intra-diffusion.

(14) $$\ln\left(\frac{C}{C_{0}}\right)=-\sqrt{\frac{t}{\tau }}$$

where

(15) $$\tau =\frac{1}{K_{d}{}^{2}}$$

and

(16) $$K_{d}=\frac{1}{\sqrt{\tau }}$$

**Figure 11:**
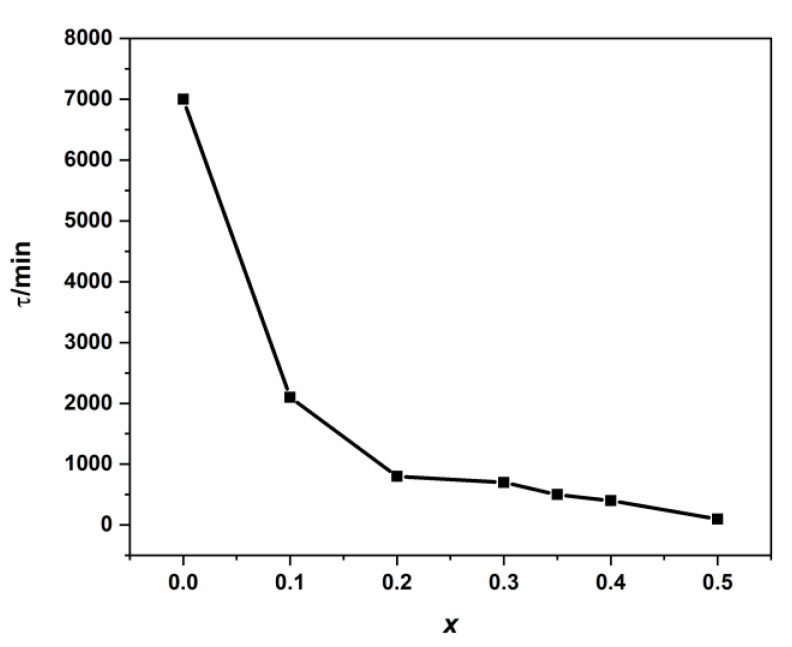
Variation of intra-diffusion time τ vs. cadmium coordination in NSFs.

At low *τ*-values, a higher intra-diffusion rate is reached (Table S2 and Figure 11). Parameter (*τ*) can be attributed as characteristic or specific time for the intra-diffusion rate phenomenon, which has a strong causal correlation with the examined kinetic study’s specificity. 

In order to suggest an adequate empirical expression for the intra-diffusion rate constant (*K_d_*) with (*x*), we supposed that the intra-diffusion characteristic time (τ) decreases exponentially as a function of (*x*) and starting from an initial maximum value (*τ*_0_ = 7035.6 min).

(17) $$\tau =\tau_{0}e^{-\theta \left(x-x_{0}\right)}$$

where *θ* and *x*_0_ are two adjustable parameters.

**Figure 12:**
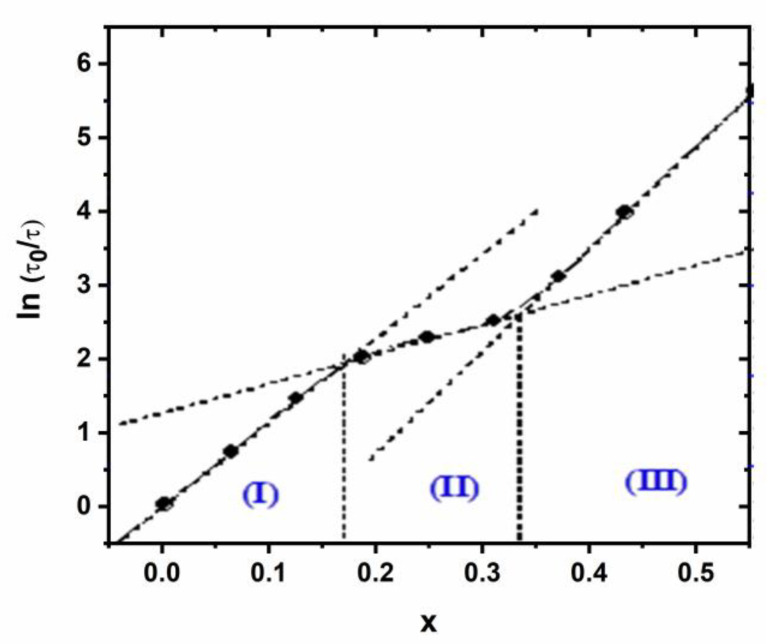
Variation of ln (τ/τ0) as a function of (x).

Moreover, to give an implicit dependence with (*x*), we have plotted, in Figure 12, the natural logarithm of the ratio (*τ*/*τ*_0_) as a function of (*x*) for which we clearly reveal the existence of the three domains with different behaviors. The quasi-perfect linearity justifies the suggested expression (Equation (17)) and permits us to delimit well the boundaries of the three fields characterized by a specific couple (*θ*,*x*_0_) (Table S3, Supplementary Information). 

Finally, considering Equations (16) and (17), we can suggest an intersecting implicit model for the intra-diffusion rate constant (*K_d_*) expressed as follows:

(18) $$K_{d}=K_{d0}e^{-\frac{\theta }{2}\left(x-x_{0}\right)}$$

where

(19) $$K_{d0}=\frac{1}{\sqrt{\tau_{0}}}$$

where the *K_d_*. Is the initial value given in Table S2 such as (*K_d_* = 0.011922 min^−0.5^) and (*τ*_0_ = 7035.6 min).

Finally, we considered that the kinetic degradation process obeys the Arrhenius law with temperature (Equation (20)) for the first pseudo-order. We can predict the relative variation of the two Arrhenius parameters ln*A*(*x*) and *Ea*(*x*).

(20) $$lnk_{1}\left(x\right)=lnA\left(x\right)-\frac{E_{a}\left(x\right)}{RT}$$

In fact, if we have preliminary data on the Arrhenius parameters (ln*A*_0_ and *Ea*_0_) at (*x* = 0), such as for the Mn_0.5_Zn_0.5_Fe_2_O_4_ ferrite nanoparticles, and we assume the global variation in Figure 8 as approximately linear versus (*x*) with a slope (*ε* = 4.9), we can differentiate Equation (20) as follows:

(21) $$\frac{\partial lnA\left(x\right)}{\partial x}-\frac{\partial E_{a}\left(x\right)}{RT\partial x}=\varepsilon$$

and the integration of the Equation (21) gives Equation (22):

(22) $$E_{a}\left(x\right)=E_{a0}+RT\left[ln\frac{A\left(x\right)}{A_{0}}-\varepsilon x\right]$$

We can then estimate the variation of the two Arrhenius parameters lnA(x) and Ea(x) for different values of (x), which can be considered as an exciting criterion of discussion and interpretation for the temperature effect.

### 3.6. Correlation between Kinetic and Optical Data 

We demonstrated, in paragraph 3.*3.* (*Optical properties*) that coordination of Cd^3+^ ion causes significant increments at direct *E*_g_ of mixed spinel ferrite samples. Cd^3+^ ion coordinated samples have band gaps 1.67, 1.68, 1.74, 1.84 and 1.87 eV of magnitude, which coincides with increasing Cd^3+^ ion ratios from *x* = 0.1 to 0.5; against 1.62 eV for Cd free spinel sample (*x* = 0).

However, the present kinetic study clearly shows that the pseudo first-order degradation rate (k_1_) increases vs. Cd coordination (*x*), and it is slightly affected by the textural properties (B.E.T. surface area) of mixed spinel ferrite samples. All the surface works in the photocatalysis process. The limiting step remains the recombination rate of the photogenerated (electron-hole) pairs rather than the adsorption of penicillin G onto the surface of the spinel ferrite samples.

**Figure 13:**
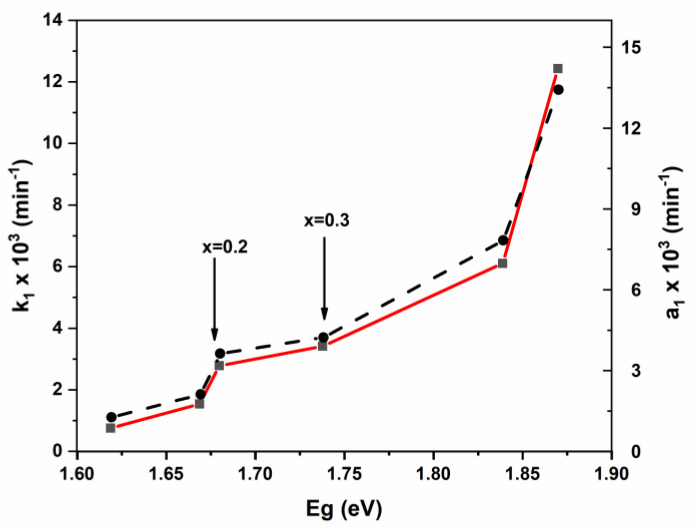
Variation of pseudo rate constant (k1) and modified pseudo-rate constant (a1) vs. bandgap energy.

To find-out a possible explanation of our results, we plotted the pseudo-first-order rates k_1_ and a_1_ (obtained from the modified model) vs. the direct bandgap energy (Eg) (Figure 13).

From Figure 13, it seems clear that the pseudo rate constant (k_1_) is bandgap dependent. The k_1_ increases slightly between 1.62 (*x* = 0) and 1.68 (*x* = 0.2). Then increases very slowly between 1.68 and 1.74 eV (*x* = 0.3). Finally, we noticed a rate jump for band gap energy higher than 1.74 eV. This finding highlights the beneficial effect of high cadmium coordination in spinel catalysts. This result could be attributed to the decrease in the recombination rate of photogenerated electron-hole pairs. Similarly, the intra-diffusion constant also shows bandgap dependence (Figure S2, Supplementary Information). (Mn_0.5_Zn_0.5_)[Cd*_x_*Fe_2-*x*_]O_4_ (x ≤ 0.05) NSFs. 

## 4. Conclusions

The effect of Cadmium metal coordination on the structural, textural, and morphological properties of spinel ferrite (Mn_0.5_Zn_0.5_)[Cd*_x_*Fe_2-*x*_]O_4_ (*x* ≤ 0.05) NSFs on the photodegradation of penicillin G (Pen G) was herein studied. The physical characterization was achieved using X-ray diffraction, Raman, UV-vis (reflectance) spectroscopies, N_2_ adsorption isotherm measurements, and S.E.M., SEM-EDX mapping, and T.E.M. allow for determining the influence of cadmium coordination on the photocatalytic response of Manganese-ZincNSFs nanostructured materials. Several catalysts were tested with different Cd coordinations (0.1, 0.2, 0.3, 0.4, and 0.5) for the photodegradation of penicillin G (Pen G) as a model molecule. The kinetic study shows that our results fit well with the pseudo-first-order model. The rate constant k_1_ increases versus Cd coordination and clearly highlights bandgap (E_g_) dependence. For *x* = 0.5, the rate constant is enhanced by a factor of 13.5 with respect to the Cd-free spinel ferrite catalyst (*x* = 0). On the contrary, the rate constant increases slightly between *x* = 0.2 and *x* = 0.3 and rapidly above *x* = 0.3. Hence, Cadmium’s presence probably induces the electric field’s formation, which decreases the recombination rate of electron-hole (e^−^, h ^+^) pairs. This finding highlights a new generation of photocatalysts with a “tuned bandgap.” Further experiments are ongoing for the treatment of industrial effluents containing penicillin G antibiotic.

## Figures and Tables

**Table 1:** Textural properties of (Mn_0.5_Zn_0.5_)[Cd*_x_*Fe_2-*x*_]O_4_ (x ≤ 0.05) NSFs.

(Mn_0.5_Zn_0.5_)[Cd*_x_*Fe_2-*x*_]O_4_	x					
	0	0.1	0.2	0.3	0.4	0.5
Surface Area (m²/g)	138	101.5	108	101	69	43
Pore Volume (cc/g)	0.180	0.190	0.210	0.196	0.150	0.086
Pore diameter (Å)	58	72	84	72	82	82

**Table 2:** C/C_0_ and Pen G degradation onto (Mn_0.5_Zn_0.5_)[Cd*_x_*Fe_2-*x*_]O_4_ (x ≤ 0.05) NSFs.

*x*	0.0	0.1	0.2	0.3	0.4	0.5
t/min	C/C_0_					
0	1.0000	1.0000	1.0000	1.0000	1.0000	1.0000
10	0.95262	0.93157	0.91825	0.87202	0.76098	0.63343
20	0.94483	0.90611	0.86885	0.82652	0.66659	0.48658
30	0.93155	0.88672	0.84257	0.81391	0.62154	0.45549
60	0.90482	0.84761	0.77303	0.71679	0.49868	0.35593
90	0.89377	0.82376	0.70142	0.67588	0.48610	0.29267
120	0.87756	0.79047	0.67476	0.61014	0.45405	0.16191
180	0.85318	0.75315	0.62379	0.55294	0.33335	0.11268
Degradation (%)	14.68	24.68	37.62	44.70	66.65	88.73

**Table 3:** Photocatalytic properties of (Mn_0.5_Zn_0.5_)[Cd*_x_*Fe_2-*x*_]O_4_ (x ≤ 0.05) NSFs.

*x*		0.0	0.1	0.2	0.3	0.4	0.5
t/min	t^0.5^/min^0.5^	ln(C/C_0_)					
0	0.0000	0.0000	0.0000	0.0000	0.0000	0.0000	0.0000
10	3.1623	−0.04854	−0.07088	−0.085286	−0.13694	−0.273	−0.4566
20	4.4721	−0.05675	−0.0985	−0.14059	−0.19053	−0.406	−0.7203
30	5.4772	−0.07091	−0.12023	−0.17130	−0.20591	−0.476	−0.7863
60	7.7460	−0.10002	−0.1653	−0.25744	−0.33297	−0.695	−1.0330
90	9.4868	−0.11231	−0.1938	−0.35465	−0.39174	−0.721	−1.2287
120	10.954	−0.13061	−0.23513	−0.39340	−0.49407	−0.789	−1.8207
180	13.416	−0.15879	−0.28349	−0.47195	−0.59251	−1.098	−2.1832
*k*_1_ × 10^3^ (min^−1^)		0.92346	1.6555	2.8425	3.4763	6.1409	12.4751
R		0.93616	0.94957	0.96295	0.96232	0.93675	0.96706

## Supplementary Materials

The following are available online at https://www.mdpi.com/article/10.3390/nano11040970/s1, Figure S1. Diffuse reflectance spectra of (Mn_0.5_Zn_0.5_)[Cd*_x_*Fe_2-*x*_]O_4_ (x ≤ 0.05) NSFs, Table S1. Kinetic parameters obtained from the modified model, Table S2. Intra-diffusion rate and corrected d factor vs. Cd coordination (x), Table S3. The boundaries of the three domains characterized by a specific couple (θ,x_0_), Figure S2: variation of Kd and Ln Kd vs. bandgap energy of mixed spinel ferrite catalysts.
