# Supplementary material for: Kinetic Modeling for Photo-Assisted Penicillin G Degradation of (Mn0.5Zn0.5)[CdxFe2-x]O4 (x ≤ 0.05) Nanospinel Ferrites
Source: Nanomaterials (Basel). 2021 Apr 9;11(4):970. doi: 10.3390/nano11040970 (PMC8070312; doi:10.3390/nano11040970)
Supplement: Supplementary file 1 [file nanomaterials-11-00970-s001.pdf]

nanomaterials

Supplementary material

Kinetic Modeling for Photo-Assisted Penicillin G Degradation of $(Mn_{0.5}Zn_{0.5})[Cd_xFe_{2-x}]O_4$ (x ≤ 0.05) Nanospinel Ferrites

Omar Alagha [1,*], Noureddine Ouerfelli [2,3], Hafedh Kochkar [2,3], Munirah A. Almessiere [4], Yassine Slimani [4], Ayyar Manikandan [5], Abdulhadi Baykal [6], Ahmed Mostafa [7], Mukarram Zubair [1] and Mohammad H. Barghouthi [1]

[1] Environmental Engineering Department, College of Engineering, Imam Abdulrahman Bin Faisal University, Dammam 31441, Saudi Arabia; mzzubair@iau.edu.sa (M.Z.); mhbarghouthi@iau.edu.sa (M.H.B.)
[2] Department of Chemistry, College of Science, Imam Abdulrahman Bin Faisal University, Dammam 31441, Saudi Arabia; Nouerfelli@iau.edu.sa (N.O.); hbkochkar@iau.edu.sa (H.K.)
[3] Basic & Applied Scientific Research Center, Imam Abdulrahman Bin Faisal University, Dammam 31441, Saudi Arabia
[4] Department of Biophysics, Institute for Research & Medical Consultations (IRMC), Imam Abdulrahman Bin Faisal University, Dammam 31441, Saudi Arabia; malmessiere@iau.edu.sa (M.A.A.); yaslimani@iau.edu.sa (Y.S.)
[5] Department of Chemistry, Bharath Institute of Higher Education and Research (BIHER), Bharath University, Chennai 600073, India; manikandana.che@bharathuniv.ac.in
[6] Department of Nanomedicine Research, Institute for Research & Medical Consultations (IRMC), Imam Abdulrahman Bin Faisal University, Dammam 31441, Saudi Arabia; abaykal@iau.edu.sa
[7] Department of Pharmaceutical Chemistry, College of Clinical Pharmacy, Imam Abdulrahman Bin Faisal University, Dammam 31441, Saudi Arabia; ammostafa@iau.edu.sa
* Correspondence: oaga@iau.edu.sa; Tel.: +966-133331682

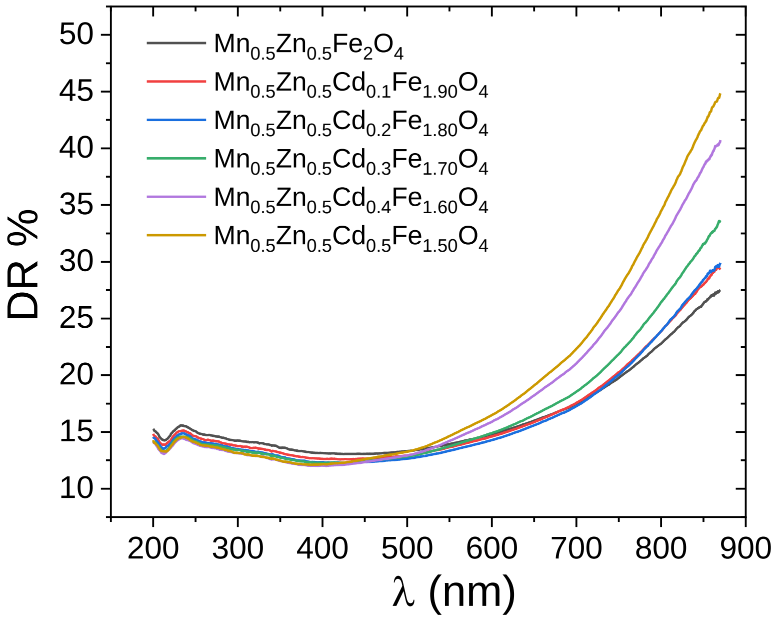

Figure S1. Diffuse reflectance spectra of $(Mn_{0.5}Zn_{0.5})[Cd_xFe_{2-x}]O_4$ (x ≤ 0.05) NSFs.

Table S1. Kinetic parameters obtained from the modified model.

x	a_1	a_2	α	*R*
	min^{-1}	min^{-2}	min^{-1}	-
0.0	0.00178325985	5.48179777e-6	0.0030740	0.977277329
0.1	0.00304330416	8.84868164e-6	0.0029076	0.983512954
0.2	0.00513746906	1.46322904e-5	0.0028482	0.99491514
0.3	0.00603187556	1.62937176e-5	0.0027013	0.988882207
0.4	0.0112121721	3.23336337e-5	0.0028838	0.969279869
0.5	0.0192415392	4.31420301e-5	0.0022421	0.981680533

Table S2. Intra-diffusion rate and corrected τ_d *factor vs Cd coordination (x).*

x	K_d	*R*	τ_d
	min$^{-0.5}$		Min
0	0.011922	0.9978	7035.6
0.1	0.021109	0.9996	2244.2
0.2	0.035656	0.9972	786.57
0.3	0.043679	0.9982	524.15
0.4	0.078955	0.9943	160.41
0.5	0.154866	0.9911	41.695

Table S3. The boundaries of the three domains characterized by a specific couple (θ,x_0).

Domain	θ	x_0	*R*
I (0 < x < 0.18)	11.565	0	0.99995
II (0.18 < x < 0.32)	4.059	-0.3407	0.99990
III (x > 0.32)	13.623	+0.1233	0.99995

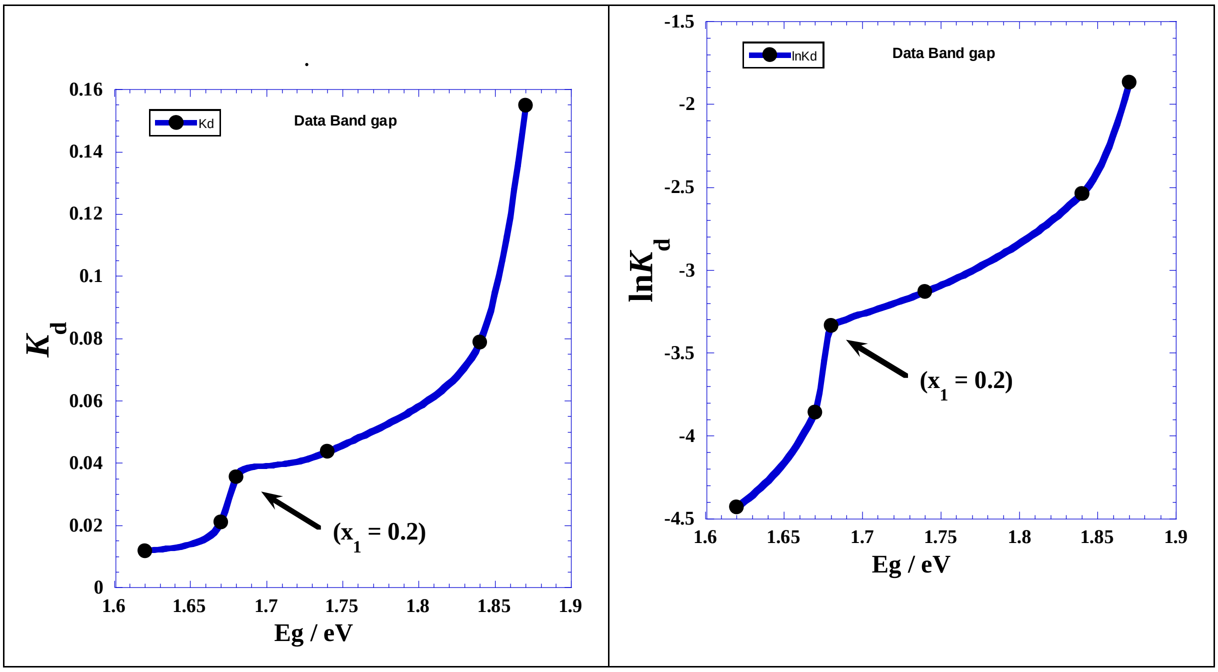

Figure S2. variation of K_d and Ln K_d vs band-gap energy of mixed spinel ferrite catalysts.
